# The Ear

**Published:** 1890-09

**Authors:** 


### THE EAR.

No oily substance, poultice or liniment should be put into the ear, because great injury is liable to be done. Warm water is the best possible, and about the only safe "wash." Do not scratch the ears with any metal ; pin-heads, hair-pins or ear-picks should be tabooed. Do not scream if an insect enters the ear ; warm water will drown it, and wash out the "remains." The ear is not nearly so liable to injury from the intruder as from frantic efforts to dislodge it. Do not put any thing cold into the ear; even cold water should be avoided, especially if there is any affection of the hearing. Do not put cotton in the ears if there is any discharge of pus from them. Use warm water as frequently as may be necessary to keep them clean, but do not force the foul matter back into the delicate machinery. If any small hard substance falls into the ear, do not attempt to "dig it out." If not readily removable, allow it to remain in quiet, and have a physician take care of it when convenient; it is not likely to do any serious harm unless tampered with. Any thing which is soluble may be washed out, with a little patience, by the use of a syringe and warm water; if not soluble it is harmless.

Deafness may sometimes be caused by an excess of ear-wax, which has become hardened, and obstructs the action of the membrane. Either have a careful hand apply warm water through a proper syringe, or a piece of cotton wadding wet with essence of peppermint may be introduced, which will dissolve and absorb the hardened wax in a few hours.

